# Percutaneous closure of aortopulmonary window using a muscular ventricular septal defect occluder. Instituto Nacional de Salud del Niño San Borja Lima-Peru. Case report

**DOI:** 10.47487/apcyccv.v6i2.465

**Published:** 2025-06-27

**Authors:** Alex Ismael Catalán Cabrera, Karen del Rosario Condori Alvino, Mónica Karem Medina Durand

**Affiliations:** 1 Instituto Nacional de Salud del Niño de San Borja. Lima, Perú Instituto Nacional de Salud del Niño de San Borja Lima Perú

**Keywords:** Heart Defects Congenital, Aortopulmonary Septal Defect, Septal Occluder Device, Cardiac Catheterization, Doppler Echocardiography, Cardiopatía Congénita, Defecto del Tabique Aortopulmonar, Dispositivo Oclusor Septal, Cateterismo Cardiaco, Ecocardiografía Doppler

## Abstract

The aortopulmonary window is a rare congenital heart defect. Isolated aortopulmonary window, without other associated anomalies, accounts for up to 25% of all cases. Surgical closure has long been, and remains, the gold standard in many cardiovascular centres. However, percutaneous closure has emerged as a viable alternative using various types of occluder devices, selected based on the morphology, size, and rims of the defect to minimise complications such as valvular interference or coronary ostial obstruction. We report the case of an infant with an isolated aortopulmonary window successfully treated with percutaneous closure using a muscular ventricular septal defect occluder device, with no complications. The patient was discharged 48 hours after the procedure.

## RESUMEN

La ventana aortopulmonar (VAP) es un defecto cardiaco congénito poco frecuente, en tanto que la VAP sin otros defectos asociados representa hasta el 25% de estos defectos, El cierre quirúrgico ha sido, y es en muchos centros cardiovasculares, el *Gold Standard*; sin embargo, el cierre percutáneo se está realizando con diferentes tipos de oclusores, tomando en cuenta el tipo de defecto, tamaño y bordes, con la finalidad de evitar complicaciones como compromiso valvular o de los *ostium* coronarios. Se presenta el caso de un paciente lactante con ventana aortopulmonar en quien se le realizó el cierre percutáneo con dispositivo oclusor de comunicación interventricular muscular, sin complicaciones, siendo dado de alta a las 48 horas del procedimiento.

## Introduction

The aortopulmonary window (APW) is a rare congenital heart defect, characterised by a septation defect between the ascending aorta and the pulmonary artery, accounting for 0.2-0.6% of all congenital heart diseases ^1^^,^^2^. It may occur in isolation or be associated, with other congenital cardiac anomalies present in 25-50% of cases, such as ventricular septal defect (VSD), tetralogy of Fallot ^3^, transposition of the great arteries, double outlet right ventricle, interrupted aortic arch ^4^, aortic atresia, and even coronary anomalies ^5^.

Clinical presentation depends on the size of the defect. Signs of heart failure are commonly observed during disease progression ^2^ and in patients older than six months, it may lead to pulmonary arterial hypertension (PAH). In such cases, cardiac catheterisation is recommended to assess the presence and severity of PAH ^6^.

Surgical repair remains the gold standard for treating this congenital defect. However, in selected cases, particularly isolated defects, percutaneous closure may offer advantages such as faster recovery, fewer complications, and earlier hospital discharge ^7^.

## Case report

We report the case of a 3-month-old male infant from Lima, Peru, with a history of full-term birth and a birth weight of 3.1 kg. He was hospitalised at 20 days of life with a diagnosis of pneumonia, during which a cardiac murmur was detected. An echocardiogram was performed, revealing an APW. Medical management was initiated with furosemide at 1 mg/kg/dose every 12 hours, spironolactone at 1 mg/kg/dose every 12 hours, and captopril at 0.5 mg/kg/dose every 12 hours. The patient was referred to the Instituto Nacional de Salud del Niño San Borja (INSN-SB).

**Figure 1:**
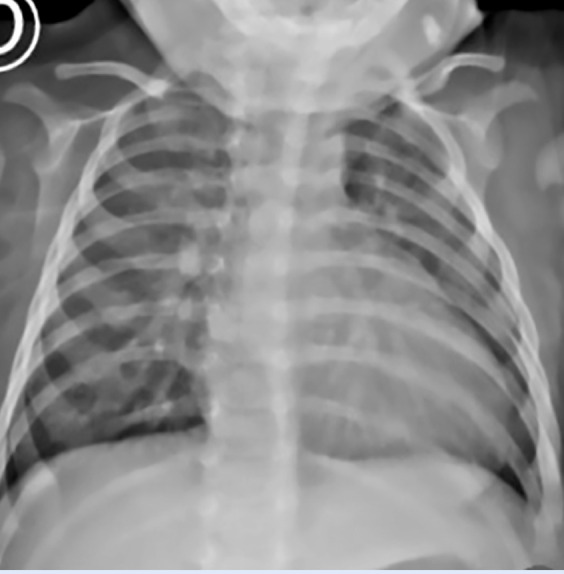
Chest X-ray on patient admission.

**Figure 2:**
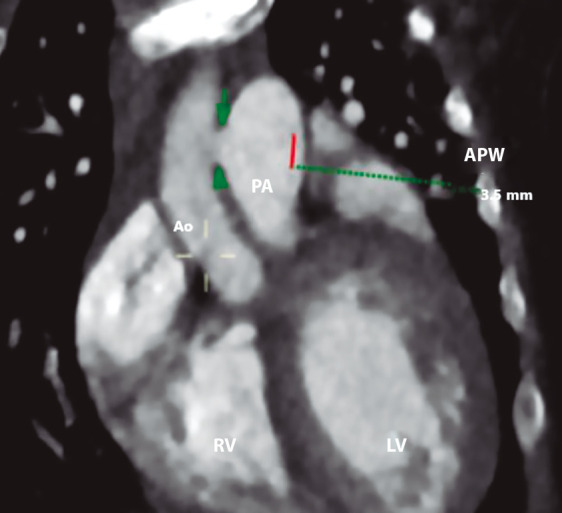
Coronal view of computed tomography showing the location of the aortopulmonary window. Delineation and measurement of the aortopulmonary window (green arrow). APW: Aortopulmonary window. Ao: Aorta. PA: Pulmonary artery. RV: Right ventricle. LV: Left ventricle.

The patient was admitted to INSN-SB at 3 months of age, weighing 5 kg, presenting with respiratory distress, poor weight gain, intercostal retractions, interrupted feeding, and oxygen saturation of 96%. Chest X-ray revealed cardiomegaly and increased pulmonary blood flow (Figure 1). Transthoracic echocardiography confirmed a type II APW measuring 3.5 mm, with left heart chamber dilation, preserved left ventricular systolic function, and low probability of pulmonary hypertension. No associated cardiac defects were identified. Cardiac CT angiography further confirmed the diagnosis of a type II APW measuring 3.5 mm, located 17 mm from both the aortic and pulmonary valve planes (Figure 2).

Once the respiratory condition was stabilised, percutaneous closure of the APW was scheduled. Under general anaesthesia, femoral arterial and venous access was obtained using 4 Fr short introducers. Unfractionated heparin was administered at a dose of 100 IU/kg. During haemodynamic evaluation, ascending aortography in right anterior oblique (RAO) 11° and cranial (CRN) 15°, as well as lateral 90° projections, confirmed the presence of a 3.5 mm APW with left-to-right shunting, located 8 mm from the aortic valve plane and 15 mm from the coronary ostia. Invasive pressure measurements revealed mild pulmonary hypertension, with a mean pulmonary artery pressure of 26 mmHg, Qp/Qs ratio of 1.6, and pulmonary vascular resistance of 0.7 Wood units/m². The aortography in RAO 11°/CRAN 15° and RAO 90°/0° projections confirmed the presence of the 3.5 mm APW (Video 1). Based on these findings, it was decided to proceed with percutaneous closure of the defect. Using a 5 Fr Judkins Right coronary catheter (JR) and a 0.035” x 260 cm hydrophilic guidewire via the venous approach, access was achieved from the main pulmonary artery to the ascending aorta, aortic arch, and descending aorta, with the guidewire positioned distally in the descending aorta (antegrade approach) (https://vimeo.com/manage/videos/1071177066).

**Figure 3:**
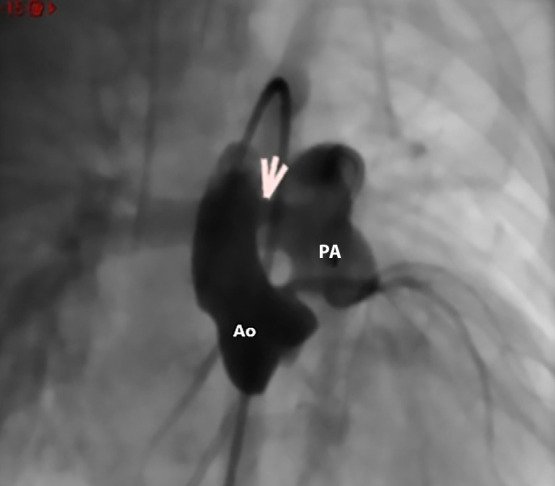
Frontal projection angiography showing the aortopulmonary window. Location of the aortopulmonary window (white arrow). Ao: Aorta. PA: Pulmonary artery.

**Figure 4:**
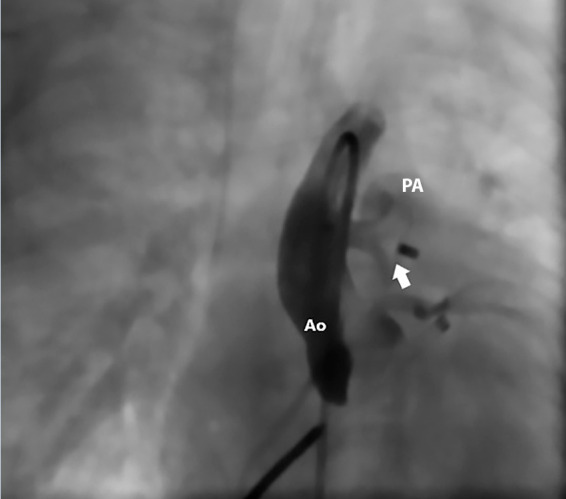
A 6 mm muscular ventricular septal defect occluder device completely sealing the aortopulmonary window. The white arrow indicates the device used. Ao: Aorta. PA: Pulmonary artery.

A 6 mm muscular VSD occluder device was advanced through a 6 Fr long sheath from the femoral vein to the ascending aorta. The first disc was deployed in the aorta, and under angiographic guidance, the device was positioned across the APW. Control angiography confirmed proper device placement with no residual shunt. No vascular or coronary obstruction was observed, and aortic valve function was preserved (Figures 3 and 4, Videos 2 and 3). Total fluoroscopy time was 19 minutes, and radiation exposure was 58.4 mGy. The patient received prophylactic antibiotic therapy with cefazolin at 50 mg/kg/dose 30 minutes prior to the procedure and completed three additional doses post-procedure.

**Figure 5:**
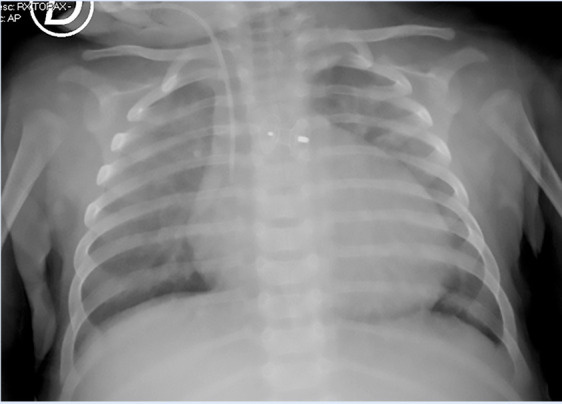
Post-procedural chest X-ray prior to discharge, showing the position of the occluder device, cardiomegaly, and pulmonary overcirculation.

The patient was discharged 48 hours after the procedure without complications. Pre-discharge echocardiography showed no residual shunt, with the muscular VSD occluder well positioned, no obstruction of the main or branch pulmonary arteries, and persistent dilation of the left heart chambers. Chest X-ray confirmed correct device positioning, mild cardiomegaly, and no evidence of pulmonary overcirculation (Figure 5). Upon discharge, the patient was prescribed furosemide at 1 mg/kg/dose every 12 hours and spironolactone at 1 mg/kg/dose every 24 hours for three months. Aspirin was not prescribed, as it is commonly used in cases of patent ductus arteriosus closure. At five-month follow-up, the patient remained clinically stable, off medication, and with normal-sized cardiac chambers.

## Discussion

APW is a rare congenital heart defect. The case presented in this report corresponds to a type II APW without associated anomalies, according to the classification of the Congenital Heart Surgery Nomenclature and Database Project of the Society of Thoracic Surgeons ^8^.

As pulmonary pressures decrease after birth, a clinical picture of pulmonary overcirculation becomes evident, often manifesting with signs and symptoms of heart failure, as observed in the patient described in this report ^6^.

Surgical repair remains the gold standard for the management of this condition ^7^, with reported mortality rates approaching 0% ^6^^-^^8^. However, in cases of isolated defects, transcatheter intervention may be considered a therapeutic alternative due to its minimally invasive nature and faster recovery. Eligibility for percutaneous closure of APW requires careful assessment of the defect’s rims to minimise the risk of valvular or coronary involvement and to prevent device embolisation ^1^^-^^9^. Trehan ^7^, in his report of three cases in infants, stated that percutaneous closure of isolated APW is both safe and feasible in this age group, and that the muscular VSD occluder may be the ideal device given its low profile, particularly when using a retrograde approach.

Ductus arteriosus occluders ^10^^,^^11^, VSD occluders ^12^, and more recently, the multifunctional Konar-MF VSD occluder ^13^^)^ have been used for percutaneous closure of APW. In the present case, we used a 6 mm muscular VSD occluder, which features two symmetrical retention discs measuring 12 mm each. The device was properly positioned, achieving complete closure of the defect without compromising adjacent structures. Closure of the APW was accomplished without complications, and the patient showed favourable short- and medium-term outcomes.

Internationally, cases have been reported in infants younger than 3 months using various devices, further supporting the feasibility of percutaneous closure for isolated APW ^12^.

In conclusion, percutaneous closure of APW is a procedure that can be successfully performed in selected cases with adequate defect rims. Careful consideration must be given to the proximity of the defect to the valvular planes and coronary arteries.
